# The Epidemiology of Prehospital Ambulance Crashes: A National Experience Across Saudi Red Crescent Authority

**DOI:** 10.7759/cureus.42049

**Published:** 2023-07-17

**Authors:** Yousef Mohammad Alsofayan, Fahad S Alhajjaj, Jalal M Alowais, Fahad Saad M Alsuhaymi, Ameera Abdullah Almutairi, Mohammed K Alsharef, Sara Abdullah Alenazi, Yara Ibrahim S Alsadan, Safia Ali M Alshangiti, Abdulmajeed Faisal A Albalawi

**Affiliations:** 1 Decision Support Unit, Saudi Red Crescent Authority, Riyadh, SAU; 2 Emergency Medicine, Unaizah College of Medicine and Medical Sciences, Qassim, SAU; 3 Surgery, College of Medicine, Imam Mohammad Ibn Saud Islamic University, Riyadh, SAU; 4 Emergency Medicine, King Fahad Specialty Hospital, Tabuk, SAU; 5 Emergency Medicine, Ministry of National Guard - Health Affairs, Riyadh, SAU; 6 Clinical Sciences, Princess Nourah Bint Abdulrahman University, Riyadh, SAU; 7 Radiology, Maternity and Children's Hospital, Tabuk, SAU

**Keywords:** emergency medical services, accident, traffic, ambulance crash, ambulances

## Abstract

Introduction

Road traffic injuries (RTIs) have a significant impact on the healthcare system as well as the global economy. RTIs involving ambulance crashes not only cause delays in patient transfers but also endanger ambulance occupants and other road users. Due to the rising numbers of RTIs in the Kingdom of Saudi Arabia (KSA), the country's primary provider of prehospital services the Saudi Red Crescent Authority (SRCA) has established a new Trauma Epidemiology Center (TEC) following the KSA 2030 vision.

Objective

This current study was conducted to determine the causes and management-related aspects of RTIs involving ambulance crashes in KSA. We aim to highlight the current status and guide further efforts to fill gaps in knowledge and on-ground changes to prevent ambulance crashes, enhance patient care, and reduce morbidity and mortality.

Method

This is a descriptive, retrospective cross-sectional observational study of ambulance crashes in Saudi Arabia between January 2020 and July 2022. The data was extracted from the SRCA electronic database platform.

Results

One hundred and sixty accidents were recorded between 2020 and 2022 with the highest proportion of accidents recorded in Riyadh 44.29%; followed by the Eastern Province and Makkah, 15% and 12%, respectively. Ninety percent of ambulance crashes were due to direct collisions and caused by other parties in 58% of cases. It is worth mentioning that ambulance drivers fastened their seat belts in 99% of crashes.

Conclusion

This study highlights the epidemiology and distribution of prehospital ambulance crashes in the KSA. Most crashes occurred in urban areas with a reasonable contribution of the public in such crashes. Understanding contributing factors related to the vector, driver behaviors, and the surrounding environment is crucial to guide national preventive measures and help decision-makers to implement proper corrective actions.

## Introduction

Road traffic injuries (RTIs) have a significant impact on the healthcare system as well as the global economy [1]. According to the World Health Organization (WHO), RTIs are responsible for 1.35 million deaths worldwide each year [2]. RTIs are recognized as a burden in the Kingdom of Saudi Arabia (KSA) and continue to affect healthcare systems despite significant efforts to improve traffic safety. According to data published in 2020, road traffic fatalities in KSA accounted for 9% of total fatalities, ranking the country 34th in the world [3]. Furthermore, the Ministry of Health (MoH) stated that the total number of deaths from car crashes in 2020 was 4,618. Individuals aged 19 to 30 are the most affected by the RTI burden, which has a cascading effect on population health [4].

Numerous epidemiological characteristics, such as the host, agent, and environment, as well as the three stages of prevention (pre-event, event, and post-event), based on the Haddon concept tool, can be used to determine the severity of harm resulting from collisions [5]. Crashes in general have occurred from many causes, but human driving errors are by far the most frequent. RTIs are primarily caused by driving behaviors such as speeding and disregarding traffic laws [6]. One of the most frequently reported causes is mobile phone usage while driving. Moreover, unsafe infrastructures, poor road conditions, and traffic congestion can all significantly contribute to RTIs. Weather changes, such as rain and sandstorms, also have a serious impact on car crashes [7].

Ambulance crashes are a growing source of public concern [8]. Crashes involving ambulances not only cause delays in patient transfers but also endanger ambulance occupants and other road users [9]. Ambulance crashes may contribute to civilian population injuries and fatalities, from 1996 to 2012 there were 64 fatalities and 217 injuries because of ambulance incidents that have been reported in the United States [10]. Ambulance crashes are caused by behavioral factors like over-speeding and crossing the red light. Also, physical, and mental stress which results from fatigue contributes to a drop in the level of paramedics emergency medicine providers' performance. In addition, there is a significant correlation between the use of sirens and red lights with ambulance crashes [11].

Road traffic accidents (RTAs) can be avoided, and governments must take comprehensive steps to address road safety. According to WHO there are proven measures to lower the risk of RTIs and deaths related. The United Nations' 2030 Agenda for Sustainable Development contains aggressive goals for reducing injuries from traffic accidents. The WHO has offered effective interventions, such as designing safer infrastructure, including road safety features in land-use and transportation planning, improving vehicle safety features, and improving post-crash health care for victims of traffic crashes. Nevertheless, enforcing key risk laws and increasing public awareness is also an important part of WHO interventions [12]. The National Highway Traffic Safety Administration and Ground Ambulance Crash (NHTSA) in the United States have adopted a proactive approach to enhance ground ambulance safety. They achieve this through various means, including collecting comprehensive crash data, conducting research, collaborating with national organizations and Federal partners, fostering consensus on promoting safe emergency medical services (EMS) systems and supporting national projects. These efforts hold the potential to significantly reduce the number of ambulance crashes [13]. The Ministerial Committee of Traffic Safety (MCTS) of the KSA aims to improve the traffic safety system to reduce the number of crashes, deaths, injuries, and the direct and indirect social and economic effects. There has been a substantial improvement in traffic safety indicators in the country. The efforts made by the MCTS members during the year 2021 have achieved results that exceeded the planned expectations in comparison to the year 2020. For example, the goal set for road traffic crash injuries in 2021 per 100,000 individuals was 88.16 injuries, and the actual rate was 72.23 injuries, meaning that the goal was achieved to a greater than 99%. Although the numbers of RTIs in KSA are still high [14], the Saudi Red Crescent Authority (SRCA) the country's primary provider of prehospital services has established a new center for trauma epidemiology named the Trauma Epidemiology Center (TEC) following the KSA 2030 vision (https://www.vision2030.gov.sa/v2030/vrps/hstp/). It focuses on improving traffic safety by identifying high-risk populations, providing current traffic safety-related scientific resources, and lowering the mortality rate from vehicle incidents. Addressing country RTIs is demanding; thus, the new center has created five main strategies to combat it. Starting with developing trauma surveillance and registries, supporting the country’s trauma-related scientific research, providing experts in the field of trauma epidemiology, assisting trauma prevention programs based on scientific documentation strategies, and encouraging community involvement in trauma prevention [15].

To better understand the process of this burden and to propose solutions to improve patient care and lower morbidity and mortality, this paper sought to identify the distribution and epidemiological characteristics of ambulance crashes across the nation.

## Materials and methods

This is a descriptive, retrospective cross-sectional observational study of all the ambulance crashes in Saudi Arabia between January 2020 to July 2022. The data was extracted from the electronic database platform of SRCA. The SRCA is a government organization that offers EMS to Saudi citizens and all other residents across KSA. It dedicates its services to providing urgent care and transportation for sick patients and victims of accidents, disasters, and other incidents. All ambulance crash data were captured into an electronic platform by paramedics driving ambulances which includes patient demographics, type of medical conditions, patients’ status, receiving hospital information, and time (of call, transport, and reaching hospital). All data was stored in SRCA's secured central server. The data included location, accident, environmental, and vehicle information were extracted by two expert healthcare providers, filled into electronic sheets, reviewed, and approved by the primary author to eliminate the discrepancy. The data privacy was maintained in all electronic sheets by two steps verification password, and the data access was limited only for those working on the study. All ambulance crashes were included in the study after the removal of duplicated data, and there were no exclusion criteria.

The study variables include the locations of ambulance crashes in different regions of KSA. Accident information consists of causes of collision (direct collision, running a red light, runover, counterflow, roadway surface), fault percent committed by each side of the reported accidents, environmental data (weather status, date, and time), and vehicles information (such as vehicle brand, model year, and the vehicle condition before and after the accident).

The study's primary outcome is to describe the distribution and epidemiological profiles of prehospital ambulance crashes in KSA. Secondary outcomes include causes of collision, and vehicle information related to ambulance crashes to prevent future incidents. Following the accident, the vehicle will be classified as either good or poor based on the criteria of taqdeer which is an integrated system for vehicle accident damage assessment (taqdeer.sa).

The study was approved by the Institutional Review Board (IRB) of the SRCA with IRB No. 22-14E on May 8, 2022. Python (www.python.org) was used for data cleaning. Data analysis was carried out using John's Macintosh Project, 16th version (JMP 16, www.jmp.com). Descriptive statistics were performed. Frequency, percentages, and numbers were used to display categorical variables.

## Results

The frequency of ambulance accidents in different regions of KSA varies widely. Riyadh occupies the first place regarding the number of traffic accidents at 44% (n=97). In second place comes Eastern Province and Makkah with 15% (n=33) and 12% (n=26), respectively. Further distribution of ambulance crashes is shown in Table 1.

**Table 1:** Frequency and percentage of prehospital ambulance crashes across various regions in KSA during the study period. KSA: Kingdom of Saudi Arabia

Variable	Region	% (n)
Region (N=219)	Riyadh	44% (97)
	Eastern Province	15% (33)
	Makkah	12% (26)
	Madina	7% (15)
	Al-Qassim	5% (12)
	Jizan	5% (10)
	Aseer	5% (10)
	Tabuk	3% (6)
	Al Jowf	2% (5)
	Al Bahah	1% (3)
	Hail	0% (1)
	Najran	0% (1)

A total of 186 of the fleet involved in crashes were Ford Transit, while 29 ambulance vehicles were GMC Savana. The distribution of the model year of ambulances subjected to traffic accidents was 2018, 2019, and 2015 with 32% (n=66), 27% (n=56), and 14% (n=29), respectively. Eighty-nine percent (n=108) of vehicle ambulances were in good condition before the accident against 11% (n=14) that were in poor condition. On the other hand, 51% (n=84) of ambulances were in a good state after the accident versus 49% (n=81) that were out of service see Table 2.

**Table 2:** Vehicle brand, model year, and vehicle condition before and after the accident during the study period.

	% (n)
Vehicle Brand (N=216)	Ford Transit 86% (186)
	GMC Savana 13% (29)
	Toyota 1% (1)
Model Year (N=205)	2021 1% (2)
	2020 13% (28)
	2019 27% (56)
	2018 32% (66)
	2017 1% (3)
	2015 14% (29)
	2014 8% (17)
	2013 0% (1)
	2010 1% (3)
Vehicle Condition Before Accident (N=122)	Good 89% (108)
	Poor 11% (14)
Vehicle Condition After Accident (N=165)	Good 51% (84)
	Out of Service 49% (81)

Among 160 accidents recorded between 2020 and 2022 in different regions of KSA, 90% (n=144) have occurred following a direct collision, 3% (n=4) after running a red light, and 1% (n=2) because of bad road conditions. Fifty-eight percent (n=78) of accidents occurred following faults committed by drivers of the other side, whereas 33% (n=44) were due to ambulance driver fault, and in 7% (n=10) both parties were at fault. Interestingly, 99% (n=109) of ambulances drivers fastened their seatbelts during the accidents see Table 3.

**Table 3:** Potential causes for ambulance crashes, fault percent, number of drivers fastening a seatbelt.

Variable	Outcome	% (n)
Causes (N=160)	Direct Collision	90% (144)
	Running a Red light	3% (4)
	Bad Road Condition	1% (2)
	Counterflow	1% (1)
	Runover	1% (1)
	Undetermined	5% (8)
Who Is At Fault (N=135)	Other Party's Fault	58% (78)
	Ambulance Driver Fault	33% (44)
	Both at Fault	7% (10)
	Not Determined	2% (3)
Seat Belt (N=110)	Yes	99% (109)
	No	1% (1)

**Figure 1:**
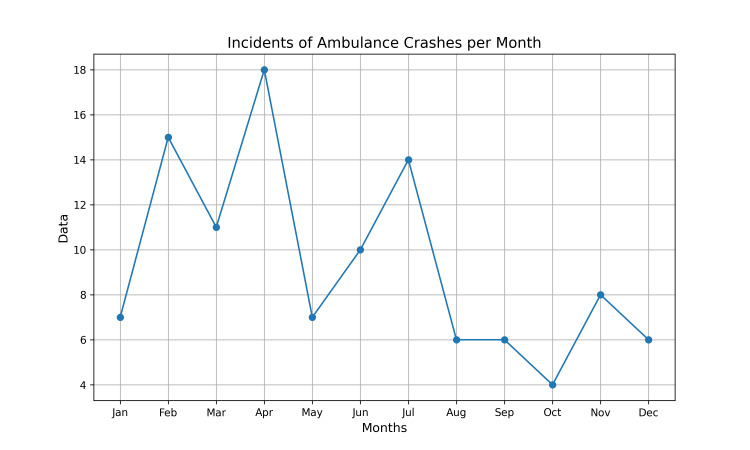
Incidents of ambulance crashes per month

**Figure 2:**
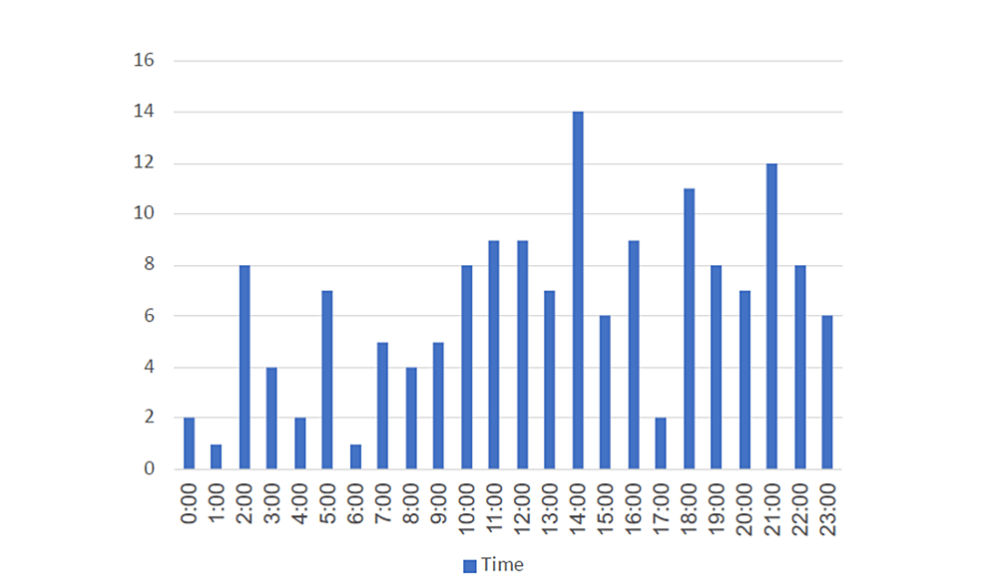
Incidents of ambulance crashes per hour

Figure 1 and Figure 2 show incidents of ambulance crashes per month and incidents of ambulance crashes per hour, respectively.

## Discussion

On several occasions, published data and literature have discussed the risk factors and causes of RTAs. Nonetheless, no regional study has demonstrated the questions regarding ambulance crash contributing factors and related present and past conditions that have resulted in an effect on ambulance crash causes thus far. There are several factors involved in emergency vehicle crashes such as driver-related, vehicle-related, and environmental-related. The driver’s risky behaviors including running red lights and counterflow are strongly associated with the urgency of emergency duty. There is a great need for the prevention of emergency vehicle crashes and to achieve this we need the epidemiology and characteristics of ambulance crashes in the region. Time pressure is one of the most dangerous features of emergency vehicle driving which is directly linked to the driver’s related risk. The urgency requirements often induce drivers to rush over the speed limit, which leads to vehicle crashes. The long working hours of drivers, sleep deprivation, fatigue, workload, irregular shifts, mental and physical stress, lack of competence and training, risky driving behavior, and overconfidence are the contributing factors that maximize the risk of crashes [10]. Studies from the USA reported similar findings and that most ambulance crashes and fatalities happened during emergency response [8]. It has been reported in our study, that the peak hour of ambulance crashes was around 2:00 in the afternoon, which is thought to be due to the rush hours, as most school, university staff, and governmental institutions employees are released around that time. Similarly, a study in the United States reported that ambulance crashes were most likely to occur between noon and 6 PM. [10]. A study in the southern region part of Saudi Arabia further confirmed our findings with the majority of RTAs occurring between 2:30 and 3:30 in the afternoon mostly due to the haste to go home, work hours are done and schools are closed [6].

This study found that the incidents of ambulance crashes were higher in the month of April followed by February and over the past three years, the month of April in the Gregorian Calendar was equivalent to the holy month of Ramadan in the Islamic Hijri Calendar which has been known for its high rates of RTAs [1,16]. The Holy Ramadan is a religious ritual with reduced working hours for employees and students. Fasting during Ramadan is associated with decreased brain performance and could lead to fatigue, impaired cognition, decline in physical performance, mood changes, and sleepiness [4,1]. Furthermore, fasting increases the incidence of hypoglycemia and is associated with an increased risk of accidents [4,5].

The primary finding of this study suggests that the capital city of Riyadh has the highest incidence of ambulance crashes, followed by the Eastern region. Makkah and Madinah, the two Islamic holiest cities, have also recorded an unprecedented incidence of ambulance crashes [1,2]. The high population and urbanization of these cities and the higher frequency of traffic could account for the number of accidents, as they are consistent with studies from other countries [17,18]. A recent study from Saudi Arabia analyzed the national data of EMS response time for RTAs and reported the capital city of Riyadh marked the highest proportion of the RTAs followed by Makkah region [16,19].

The ambulance crashes involved various vehicle models. In this background, some new ambulance car models are equipped with automatic warning and road safety systems such as high-performance speed cameras. NHTSA recently published data that advocates that “new vehicles are the safest on U.S. roads” [1]. The high number of crashes involving the Ford Transit model in the SRCA fleet can be attributed to the fact that it constitutes the majority of the fleet, thus explaining the higher incidence compared to other models.

It is important to note that the age of the vehicle may not be the determining factor in the accidents, but rather the maintenance and the use of the vehicle. Regular servicing, inspections, and repairs can help identify and address potential issues that could contribute to accidents. Our study demonstrated that a total 81 of ambulances were out of service after the crash incident, representing 49% of total crashes. This huge number will have a significant impact on the healthcare system that will delay patient transfer to and from healthcare centers [11]. Similarly, a study conducted in the USA found that 58.7% of ambulances involved in crashes became inoperable or disabled [8]. Studies suggested the use of smart health systems to manage EMS in all regions including urban and rural help manage the ambulance rotations between different areas. These systems facilitate the monitoring of the location, speed, and service maintenance of the vehicle to prevent mechanical issues and sustain the operation [20].

Planning to eliminate both environmental and host-related risk factors leading to severe accidents and critical traumas in the first place can decrease the financial and health-related burden of ambulance crashes. According to a scoping review article, out of six articles that looked at the association between road conditions and ambulance accidents stated that dry road conditions were more accident-prone than wet ones [11].

We found between the years 2020 and 2022 the most common cause of ambulance crashes was direct collision. Having said that, the importance of conducting driver background checks for ambulance operators, as well as the potential repercussion of a bad driving record is an essential aspect of maintaining a safety profile [10]. Driver’s mental and physical health is also an important consideration and long working hours contribute to fatigue and its related consequences such as ambulance crashes. A systematic review based on 100 studies suggested that <24 hours working duration is more favorable for the mental health of emergency medical staff than shifts of >24 hours [21]. It’s worth mentioning that during the COVID-19 pandemic, the number of ambulance crashes was reduced in various regions mostly due to lower traffic volume. During the curfew measures in KSA (between March and June 2020) the number of ambulance crashes was 21.

There is limited data available globally on the epidemiology and characteristics of ambulance crashes and almost scarce in the KSA. This study is a valuable contribution to designing and implementing better management strategies to minimize the number of ambulance crashes. However, our study has several limitations; this study surely does not collect all data on ambulance crashes from all the regions of Saudi Arabia. This study collected data from the years between 2020 and 2022, which also missed the number of ambulance crashes. Another important consideration which lacked is the driver’s competence and qualification.

## Conclusions

This study highlights the epidemiology and distribution of prehospital ambulance crashes in the KSA. Most crashes occurred in urban areas with higher rates in the holy month of Ramadan. Various contributing factors related to the vector and vehicle maintenance, ambulance drivers and lack of sleep, and the surrounding environment. Further studies are crucial to guide national preventive measures and decision-makers to implement proper corrective actions to reduce the impact of ambulance crashes.
